# The Landscape of Outpatient Palliative Care in Germany: Results from a Retrospective Analysis of 14,792 Patients

**DOI:** 10.3390/ijerph192214885

**Published:** 2022-11-12

**Authors:** Sven H. Loosen, Sarah Krieg, Johannes Eschrich, Mark Luedde, Andreas Krieg, Manuela Schallenburger, Jacqueline Schwartz, Martin Neukirchen, Tom Luedde, Karel Kostev, Christoph Roderburg

**Affiliations:** 1 Clinic for Gastroenterology, Hepatology and Infectious Diseases, University Hospital Duesseldorf, Medical Faculty, Heinrich-Heine-University Duesseldorf, 40225 Duesseldorf, Germany; 2 Department of Hepatology and Gastroenterology, Charité University Medicine Berlin, Augustenburger Platz 1, 13353 Berlin, Germany; 3 KGP Bremerhaven, 27574 Bremerhaven, Germany; 4 Department of Surgery (A), University Hospital Duesseldorf, Medical Faculty, Heinrich-Heine-University Duesseldorf, 40225 Duesseldorf, Germany; 5 Interdisciplinary Center of Palliative Medicine, University Hospital Duesseldorf, Medical Faculty, Heinrich-Heine-University Duesseldorf, 40225 Duesseldorf, Germany; 6 Department of Anesthesiology, University Hospital Duesseldorf, Medical Faculty, Heinrich-Heine-University, 40225 Duesseldorf, Germany; 7 Epidemiology, IQVIA, 60549 Frankfurt, Germany

**Keywords:** cancer, heart failure, SAPV, diagnosis, palliative medicine, outpatient

## Abstract

Background: Palliative care comprises multiprofessional, integrated, person-centered healthcare services for patients and their families facing problems related to progressive or advanced diseases and limited life expectancy. Although non-oncology patients’ needs are similar to those of tumor patients, they are often underestimated. The purpose of our study was to investigate the actual utilization of palliative care services in Germany, especially in the outpatient setting. Methods: Using the IQVIA Disease Analyzer database, a total of 14,792 outpatients from 805 primary care practices in Germany with documented palliative care and related diagnosis between 2018 and 2021 were analyzed. Proportions of different diagnoses among patients receiving outpatient palliative care were stratified by gender and different age groups. Results: The most common underlying diagnosis for outpatient palliative care was cancer (55%), followed by heart failure (16%) and dementia (8%), with age- and sex-specific differences found in the proportion of diagnoses for utilization. While the relative proportions of cancers decreased with age (87% in the 18- to 50-year-old age group versus 37% in the 80-plus age group), the proportion of palliative care related to heart failure increased in the older population (2% in the 18- to 50-year-old age group versus 25% in the 80-plus age group). Conclusions: This study provides an overview of the situation of outpatient palliative care in Germany and shows age- and gender-specific trends regarding the underlying medical diagnoses. Based on these data, palliative care should be adapted to current demographic developments.

## 1. Introduction

Patients with symptom burden who are seriously ill and dying are entitled to specialized palliative care. Palliative care aims to alleviate the consequences of an illness (palliation) when there is no longer any prospect of a cure [1,2]. Palliative care can be provided at home, in hospital, in a nursing home or in a hospice [3,4,5]. Outpatient palliative care (OPC) means that patients receive both medical and nursing care in their familiar home environment. This often enables them to die with dignity and as little symptom burden as possible [3,4,5]. The expansion of OPC meets the desire of many seriously ill patients to remain in their home environment and close to their relatives [6,7]. Effective 1 April 2007, in Germany, the legislature included specialized OPC as an individual entitlement to benefits in the German Social Code Book V [8,9]. Since then, insured persons are also entitled to this new form of care, the aim of which is to make it possible for patients with particularly complex care needs to receive care and support at home. Specialized OPC (SAPV) services—in addition to general OPC—maintain, promote and improve the quality of life and self-determination of palliative patients as far as possible and enables them to live in dignity until death in their familiar surroundings, within an ambulant setting [8]. In contrast to general OPC, specialized OPC makes the deployment of a specialized palliative care team necessary—temporarily or permanently [10,11,12]. It takes place within the framework of a care structure exclusively geared to palliative care. This involves, in particular, specialized palliative medical and nursing consultation and/or care, including the coordination of necessary care services up to comprehensive, individual support management. Multiprofessionalism, 24-h accessibility seven days a week, and specialist status (through continuing education and experience) of individual providers primarily practicing in palliative care are essential. However, only a (small) proportion of all seriously ill and dying people require this special form of care, while the majority of patients is receiving general OPC by their respective general practitioner [10,11,12].

Today, there are about 1500 outpatient hospice services, about 250 inpatient hospices for adults and 19 inpatient hospices for children, adolescents and young adults, about 340 palliative care units in hospitals, four of them for children and adolescents [13]. There are 403 specialized OPC (SAPV) teams in Germany [14]. By 2021, 14,620 physicians had completed additional training in palliative medicine [15]. More than 120,000 people are involved on a voluntary, civic and full-time basis and support the work for seriously ill and dying people. In this respect, an extensive infrastructure has been established in recent years to ensure palliative care in the outpatient setting.

The purpose of this study was to provide an impression of the burden of palliative care in the outpatient setting on the public health system. We aimed to increase awareness of and improve access to palliative care by identifying which persons are most likely to use OPC, considering diagnosis, age, and sex.

## 2. Materials and Methods

### 2.1. Data Source

This study represents a retrospective database cross sectional study based on the IQVIA Disease Analyzer (DA) database, which contains case-based information including demographic data, diagnoses, and prescriptions information provided by office-based physicians (GPs and specialists) in Germany. The quality of the data is regularly assessed by IQVIA on a number of criteria (e.g., completeness of documentation and linkage between diagnoses and prescriptions). It has been previously found that the panel of practices included in the DA database is representative for the general and specialized practices in Germany [16]. The database used includes only anonymized data in compliance with the regulations of the applicable data protection laws. German law allows the use of anonymous electronic medical records for research purposes under certain conditions. According to this legislation, it is not necessary to obtain informed consent from patients or approval from a medical ethics committee for this type of observational study that contains no directly identifiable data. Because patients were only queried as aggregates and no protected health information was available for queries, no Institutional Review Board approval was required for the use of this database or the completion of this study.

### 2.2. Study Population

This study included individuals (18 years or older) in 805 general practices (GP) with at least one documentation of palliative support between 1 January 2018 and 31 December 2021. Palliative support was considered using billing numbers according to the appropriate value measurement (German: EBM) and the fee regulations for doctors (German: GOÄ) including 03370, 03371, 03372, 03373. The day of the first palliative support documentation was considered the index date.

### 2.3. Study Outcomes

The main outcome of this study were proportions of different diagnoses among patients receiving palliative outpatient care. These diagnoses included cancer (ICD-10: C00-C97), heart failure (ICD-10: I50), coronary heart disease (ICD-10: I25), stroke (ICD-10: I60-I64), renal failure (ICD-10: N18, N19), chronic obstructive pulmonary diseases (ICD-10: J44), liver cirrhosis (ICD-10: K74, K70.3), dementia (ICD-10: F00-F03, G30), other neurological disorders (Parkinson’s disease, ICD-10; G20, G21; multiple sclerosis, ICD-10: G35; spinal muscular atrophy and related syndromes; ICD-10: G12). For cancer diagnoses, the most frequent cancer sites documented were shown. All analyses were conducted for all patients in total as well as five age groups (18–50, 51–60, 61–70, 71–80, >80 years), and women and men separately. This study used descriptive statistics. Analyses were carried out using SAS version 9.4 (Cary, NC, USA: SAS Institute Inc.).

## 3. Results

### 3.1. Patient Characteristics

A total of 21,798 individuals receiving palliative care were identified from the DA database within the study time period. The mean age at index date (SD) was 75.0 years (15.7 years). 55.5% of patients were female. Among these, information on the underlying medical diagnosis was available for 14,792 patients. In this subgroup, mean age (SD) was 77.4 years (12.4 years) with a female proportion of 54.0%.

### 3.2. Most Frequently Coded Diagnoses among Patients Receiving Palliative Care 

Table 1 summarized the distribution of underlying diagnoses in total as well as stratified by sex. More than half of patients undergoing palliative care (55%) had a diagnosis of cancer (Table 1). This proportion was numericaly even higher among female patients (60%) compared to male patients (51%). The second most common diagnosis leading to palliative care was heart failure (HF), which was coded in 16% of cases. The proportion of HF patients was numericaly higher among male (19%) compared to female (13%) palliative care patients. Dementia was the third most common coded diagnosis in palliative care patients, accounting for 8% of cases, with a higher proportion of dementia cases in female patients (10%) than in males (6%). Other diagnoses necessitating palliative care were coronary heart disease (CHD) (7%), renal failure (4%), stroke (4%), and COPD (3%, Table 1).

The relative proportion of cancer diagnoses decreased with age from 87% in the age group between 18 and 50 years to only 37% in the age group above 80 years (Table 1). In contrast, HF had a proportion of only 2% in the age group between 18 and 50 years, which increased to 25% among patients above 80 years. In this age group of patients above 80 years, dementia (13%) and CHD (10%) were also relative frequent (Table 1).

### 3.3. Cancer-Associated Palliative Care and Tumor Entities

Among all 8138 cancer patients, digestive organ cancer (28%) was the most prevalent tumor entity followed by respiratory tract cancer (18%) and lymphoid and hematopoietic tissue cancer (12%, Table 1). Among cancers of the digestive organs, colorectal cancer (10%) and pancreatic cancer (7%) had the highest proportion.

In men, digestive organ cancer (30%), respiratory tract cancer (21%), and male genital organ cancer (15%) represented the three most frequent tumor entities. In women, female breast or genital organ cancer (29%), digestive organ cancer (27%), and respiratory tract cancer (16%) were most commonly diagnosed (Table 2). With respect to different age groups, the proportion of digestive organ cancer varied between 23% in the age group between 18 and 50 years and 31% in age group above 80 years. The proportion of respiratory tract cancer varied between 10% in the age group between 18 and 50 years and 27% in age group between 61 and 70 years (Table 2).

## 4. Discussion

Palliative care is indicated to patients when there is no longer a chance of a cure and life expectancy is limited [17]. Although the concept of palliative care originated in cancer medicine, it is increasingly gaining importance in the treatment of other progressive chronic diseases [18,19]. Elderly people with cardiovascular diseases, dementia, frailty or multimorbidity represent the largest patient groups in the outpatient sector. Based on data from the DA database from 2018 to 2021 involving a total of 14,792 individuals from 805 general practices, our study provides a good overview of which individuals are seeking palliative care in the outpatient setting based on diagnosis, age, and gender. In summary, we confirmed that palliative care is still most common among tumor patients. However, our data showed that palliative care for chronic non-cancerous diseases is becoming increasingly important. Interestingly, the proportion of cancer diagnoses in palliative care decreased with age, whereas HF gained importance as a reason for palliative care in the elderly patients. The new “2022 AHA/ACC/HFSA Guideline for the Management of Heart Failure” emphasizes for the first time that palliative care should be an integral part of early integration throughout the disease course and at all stages of HF. According to the guideline recommendation, palliative care should be started in the early stages of the disease and continued until the terminal stage, and even until the bereavement of the relatives after death [20]. Early integration of palliative and supportive approaches into the care of patients with HF has been shown by several studies to improve various processes of care and patient outcomes [20]. For example, as the disease progresses, increasingly important decisions are made about the use and discontinuation of potentially life-sustaining therapies such as the use of inotropics, implantable cardioverter-defibrillators (ICD), mechanical circulatory support (MCS) or renal replacement therapy (RRT). In contrast, failure to proactively address issues such as deactivation of ICD and left ventricular assist device (LVAD) therapies can lead to end-of-life suffering [21,22]. The guideline specifies that formal palliative care consultation in HF may be particularly helpful in patients with refractory symptoms, serious medical decisions, as well as multimorbidity, frailty, or cognitive impairment. Remarkably, 1–2% of the adult population in high-income countries is affected by HF with at least 5% of this group suffering from an advanced stage with a high symptom burden [23,24]. Due to the increasing life expectancy of the population and the improved treatment options for HF with longer disease duration, the number of patients with HF is expected to rise in the coming years. Notably, it has been demonstrated in previous studies that patients with HF could have similar symptoms and a similar poor prognosis as cancer patients [25,26]. While cancer patients use to show a steady progression with a relatively clearly definable terminal phase, patients with non-malignant diseases, such as HF, often have a slower progression over longer periods of time [27]. This may result in patients with HF presenting late or not at all for palliative care. Palliative intervention in HF patients has been shown in previous studies to result in significant decreases in symptom burden and hospitalization rates as well as improvements in quality of life [28,29,30,31].

Furthermore, looking at the most common underlying diagnoses in our study, we see that dementia is becoming increasingly important in palliative care, particularly in the group of patients aged ≥ 80 years. It is estimated that the number of dementia cases will continue to increase in the future due to the aging population [32]. A particular challenge for patients with advanced dementia is difficult communication in assessing and managing symptoms such as pain and in establishing their wishes for end-of-life care, which could lead to delayed integration into palliative care.

Our results support the data of Gothe et al., who, similar to our study, analyzed the use of palliative care at the national level based on a large German health data set with a total of 14,522 patients. The authors estimated an annual number of 410,000 patients with a palliative care condition, noting that both incidence and prevalence increased sharply with age. At the same time, the authors’ findings suggest an unmet need for palliative care, particularly among younger and middle-aged people. This was attributed to the dominance of curative treatment attempts for life-threatening diseases, even when palliative care would have already been indicated [33] Similar to our study, Gothe et al. considered the full spectrum of palliative care-relevant conditions. However, in addition to the study by Gothe et al., our study is distinguished in that the main diagnosis indicated for palliative care has also been stratified by age. This additional analysis allowed us to get a sense of the extent to which the patient’s disease profile changed with age when palliative care was considered.

Matching our study’s findings, Gothe and colleagues showed an average age of patients receiving palliative care of 77 years [33]. Demographic change, accompanied by steadily increasing life expectancy, will not only bring increasing multimorbidity, but will also require greater demand for palliative care [34]. Consequently, palliative care services have to be continuously improved and expanded, especially addressing the diverse needs of the elderly population in the future. This will require expertise and good interdisciplinary and interprofessional teamwork among specialists such as geriatricians, oncologists, cardiologists, palliative care physicians, nurses, pharmacists, psychologists, physiotherapists, social workers, dietitians, nurses, speech therapists, and chaplains at various stages of illness.

Another aspect of our study was to compare the proportion of patients receiving palliative care according to sex. Thereby, we observed that palliative care is more frequently used by female than by male patients. Our finding of a gender gap in palliative care utilization is largely consistent with the literature on gender differences [35,36,37,38,39]. These gender differences may be explained by role socialization. Therefore, social norms in many societies around the world are more likely to allow women to express feelings, report symptoms, and not see social support as a sign of weakness.

Comparing the overall admittance to palliative care of patient with different cancer entities, it was highest for patients with digestive organ cancer, followed by respiratory tract cancer, and lymphoid and hematopoietic tissue cancer. Interestingly, gender differences were found, which could be attributed to the different gender-specific cancer prevalences. Accordingly, the most common cancer in men is prostate cancer, followed by lung cancer and colorectal cancer; in women, breast cancer has the highest prevalence, followed by colorectal cancer and lung cancer.

However, some limitations have to be acknowledged when interpreting the study results. First, our study is descriptive in nature and subject to the inevitable limitations of a longitudinal and retrospective analysis of a large database. Secondary data analyses such as the present study are usually limited by the incompleteness of the underlying data. All diagnoses were documented with ICD-10 codes, which could potentially lead to misclassification and undercoding of certain diagnoses. For example, for some coded palliative care treatments, no underlying diagnosis was documented. It should also be noted that our study only displayed the diagnoses that the primary care physician indicated as the main reason for palliative care, but not the patients’ concomitant diagnoses (e.g., diabetes, hypertension, depression, and others), although many patients were comorbid. Furthermore, our data focused specifically on underlying diagnoses, age, and sex. In contrast, there was no information on symptom burden (e.g., dyspnea, anxiety, delirium), disease stage, or sociodemographic characteristics that would have allowed more detailed analyses. In this study the first palliative care documentation was considered the index date. As consequence, patients may be 1–2 years older on the end of the follow-up, but this has no effects on their sex or palliative diagnoses. Nevertheless, the IQVIA DA database used for the analyses in this study has been used extensively [40,41,42] and has proven its validity [16]. Finally, the major strengths of our study are the large number of practices and patients included and the use of data that are representative of general and specialist practices in Germany.

## 5. Conclusions

Taken together, our data highlight that palliative care is not limited to tumor patients, but increasingly includes other chronic, non-curable diseases. The percentage of non-tumor diseases in palliative care is age-dependent and is increasing in the older population. Consequently, demographic changes accompanied by continuously increasing life expectancy not only brings increasing multimorbidity, but will also require a greater number of palliative care services. Non-tumor related chronic diseases remain underrepresented and should receive greater access to palliative care. Our findings should form the basis for prospective studies in this area to further improve the integration of chronically ill patients into palliative care.

## Figures and Tables

**Table 1:** Underlying medical diagnoses of patients receiving palliative care in Germany between 2018 and 2021 stratified by sex and age.

Average Number of Patents per GP Practice								
Diagnosis	Total	Age 18–50	Age 51–60	Age 61–70	Age 71–80	Age > 80	Men	Women
Cancer	10.11	0.48	1.28	2.17	2.93	3.25	5.07	5.03
Heart failure	2.95	0.01	0.06	0.16	0.50	2.22	1.07	1.88
Dementia	1.52	0.00	0.02	0.05	0.29	1.16	0.53	0.99
Coronary heart disease	1.35	0.01	0.03	0.12	0.31	0.87	0.66	0.69
Renal failure	0.78	0.01	0.02	0.06	0.13	0.55	0.32	0.46
Status post stroke	0.72	0.01	0.04	0.05	0.16	0.46	0.29	0.42
COPD	0.58	0.01	0.05	0.13	0.15	0.24	0.30	0.28
Other neurological diseases	0.27	0.01	0.02	0.04	0.08	0.11	0.14	0.13
Liver cirrhosis	0.09	0.00	0.02	0.02	0.02	0.02	0.06	0.03
**Proportion of Patients in %**								
**Diagnosis**	**Total**	**Age 18–50**	**Age 51–60**	**Age 61–70**	**Age 71–80**	**Age >80**	**Men**	**Women**
Cancer	55.0%	86.8%	82.9%	77.5%	63.9%	36.6%	60.1%	50.7%
Heart failure	16.0%	1.6%	3.9%	5.6%	10.9%	25.0%	12.7%	18.9%
Dementia	8.3%	0.0%	1.5%	1.8%	6.3%	13.0%	6.3%	10.0%
Coronary heart disease	7.3%	1.8%	1.9%	4.4%	6.9%	9.8%	7.8%	6.9%
Renal failure	4.3%	2.7%	1.3%	2.1%	2.9%	6.2%	3.8%	4.6%
Status post stroke	3.9%	2.5%	2.3%	1.7%	3.5%	5.1%	3.5%	4.3%
COPD	3.2%	1.6%	3.2%	4.6%	3.3%	2.7%	3.5%	2.8%
Other neurological diseases	1.5%	2.2%	1.6%	1.6%	1.7%	1.3%	1.7%	1.3%
Liver cirrhosis	0.5%	0.9%	1.4%	0.7%	0.5%	0.2%	0.7%	0.4%

**Table 2:** Proportion of different tumor entities among cancer patients receiving palliative care in Germany between 2018 and 2021 stratified by sex and age.

Diagnosis	Total	Age 18–50	Age 51–60	Age 61–70	Age 71–80	Age >80	Men	Women
N (patients with cancer)	8138	388	1030	1743	2358	2619	4085	4053
Lip, oral cavity and pharynx	4%	4%	7%	6%	3%	2%	5%	2%
Digestive organs	28%	23%	25%	27%	29%	31%	30%	27%
*Stomach*	*3%*	*5%*	*3%*	*3%*	*3%*	*4%*	*4%*	*3%*
*Colorectal*	*10%*	*8%*	*8%*	*9%*	*10%*	*13%*	*11%*	*10%*
*Liver*	*3%*	*2%*	*2%*	*3%*	*3%*	*3%*	*4%*	*2%*
*Pancreas*	*7%*	*4%*	*6%*	*7%*	*9%*	*7%*	*7%*	*8%*
*Other digestive organs*	*5%*	*5%*	*5%*	*4%*	*4%*	*5%*	*5%*	*4%*
Respiratory organs	18%	10%	22%	27%	21%	11%	21%	16%
Skin	5%	5%	3%	2%	4%	8%	5%	4%
Female breast or genital organs	14%	25%	16%	13%	13%	15%		29%
Male genital organs	8%	2%	3%	7%	8%	11%	15%	
Urinary tract	5%	3%	5%	4%	6%	7%	7%	4%
Brain	3%	10%	6%	3%	2%	1%	3%	3%
Lymphoid and hematopoietic tissue	12%	13%	10%	9%	12%	14%	11%	13%
Other cancer sites	2%	6%	2%	1%	3%	1%	2%	3%

## Data Availability

The datasets used and/or analyzed during the current study are available from the corresponding author on reasonable request.
